# The Viral Janus: Viruses as Aetiological Agents and Treatment Options in Colorectal Cancer

**DOI:** 10.3389/fcimb.2020.601573

**Published:** 2021-01-07

**Authors:** Christopher J. R. Turkington, Ambarish C. Varadan, Shea F. Grenier, Juris A. Grasis

**Affiliations:** ^1^ School of Natural Sciences, University of California Merced, Merced, CA, United States; ^2^ Department of Biology, San Diego State University, San Diego, CA, United States

**Keywords:** virus, bacteriophage, cancer, colorectal, disease dynamics, bacteriophage therapy, oncolysis, microbiome

## Abstract

In recent years, our understanding of the importance of microorganisms on and within our bodies has been revolutionized by the ability to characterize entire microbial communities. No more so is this true than in cases of disease. Community studies have revealed strong associations between microbial populations and disease states where such concomitance was previously absent from aetiology: including in cancers. The study of viruses, in particular, has benefited from the development of new community profiling techniques and we are now realising that their prominence within our physiology is nearly as broad as the diversity of the organisms themselves. Here, we examine the relationship between viruses and colorectal cancer (CRC), the leading cause of gastrointestinal cancer-related death worldwide. In CRC, viruses have been suggested to be involved in oncogenesis both directly, through infection of our cells, and indirectly, through modulating the composition of bacterial communities. Interestingly though, these characteristics have also led to their examination from another perspective—as options for treatment. Advances in our understanding of molecular and viral biology have caused many to look at viruses as potential modular biotherapeutics, where deleterious characteristics can be tamed and desirable characteristics exploited. In this article, we will explore both of these perspectives, covering how viral infections and involvement in microbiome dynamics may contribute to CRC, and examine ways in which viruses themselves could be harnessed to treat the very condition their contemporaries may have had a hand in creating.

## Introduction

Our bodies are host to a fascinatingly complex community of microorganisms, termed the microbiome. Each of us contains a unique mix of viruses; bacteria; archaea; and eukaryotes, such as fungi, protists, and nematodes. From birth to death, the constituents found within our microbiomes, and how they are configured, are interwoven within nearly every facet of our existence. This is particularly true of organisms within the gastrointestinal tract, where microorganism density is greatest (Lynch and Pedersen, 2016; Sender et al., 2016). Dietary processes, such as digestion and nutrient absorption, all rely upon microorganisms to function normally (Conlon and Bird, 2015; Basolo et al., 2020). However, so do non-dietary processes, including cell proliferation and angiogenesis (Reinhardt et al., 2012; Ijssennagger et al., 2015). Critical traits from our metabolic profiles, immune responses, to even our moods are all shaped by microbes in the gut (Turnbaugh et al., 2006; Steenbergen et al., 2015; Spencer et al., 2019). Yet, their paramountcy in health also gives way to a reciprocal role in disease.

Microbial-associated diseases can arise from several origins. For example, as infections, the presence or abundance of a pathogenic organism can directly lead to the production of a disease state [e.g. *Vibrio cholerae* in cholera; Baker-Austin et al. (2018)]. Alternatively, more complex population-level changes can occur that lead to disease. Here, alterations in the taxonomic composition of the microbiome away from that found during the “healthy” state lead to disease. This can involve the loss of important commensal organisms, depleting the functional pool of the microbiome, reducing its ability to support normal physiological processes (Levy et al., 2017; Durack and Lynch, 2019). Non-communicable disorders, such as obesity, hypertension, and rheumatoid arthritis, have been linked to changes in microbial populations (Mar Rodríguez et al., 2015; Zhang et al., 2015; Li et al., 2017).

Much of the focus on gastrointestinal microbiome-associated disease has been towards its bacterial component, yet, viruses are also important drivers of disease. Viruses of eukaryotes, those that infect our cells (or other eukaryotic organisms, e.g. fungi), can considerably impact health; most notably as infectious agents. Norovirus, for instance, is the leading cause of foodborne gastroenteritis worldwide (Kirk et al., 2015). There is also growing evidence implicating viruses in conditions involving bacterial community changes. Viruses of prokaryotes, termed bacteriophages, are the most abundant viruses within the human gastrointestinal tract, and regulate bacterial populations across ecological niches (Wigington et al., 2016; Argov et al., 2017; Shkoporov and Hill, 2019). Conditions such as Crohn’s disease and ulcerative colitis, are believed to have a marked bacteriophage component in their aetiology (Norman et al., 2015).

One set of conditions that have long held an association with viruses are cancers. Viral involvement in cancers can be seen across the body, where they contribute to a significant proportion of incidence. In 2018, around 2.2 million cancers worldwide (~13% of all cancers) were caused by carcinogenic infections: the majority attributed to viruses (de Martel et al., 2020). The remainders of infection-associated cases are primarily linked to bacterial activity (e.g. *Helicobacter pylori* in gastric cancer). Given what we are beginning to understand about the importance of bacteriophages in bacterial-associated diseases, such as their involvement in driving immune dampening and selecting for pathogenic bacterial phenotypes (Hosseinidoust et al., 2013; Sweere et al., 2019), it is not implausible that some of these cases, too, may have a viral relation. In colorectal cancer (CRC), both direct and indirect involvement of viruses in disease has been suggested. Numerous studies have indicated the presence of oncogenic viruses within tumors, including viruses that have been linked to other cancers (Chen et al., 2015), while there is also evidence linking bacteriophages to CRC through their interactions with other members of the gastrointestinal microbiome (Emlet et al., 2020).

CRC encompasses both cancers of the colon and rectum. Cancer of the colon is the most common of the two, though incidence rates can differ based on factors such as gender, race, and age (Siegel et al., 2020a). The majority of cases are sporadic (~60–65%), but can be influenced by hereditary factors; either linked to a prior familial occurrence or an inherited cancer syndrome (Keum and Giovannucci, 2019). While CRC is the third most common form of cancer globally, it is responsible for the second-highest level of cancer-related death worldwide (Bray et al., 2018). Despite seeing reductions in the total numbers of CRC cases in many countries in the last decade, some countries are seeing rates of decline slowing, including the US, in large part due to increases in the incidences of early-onset CRC (cases in individuals < 50 years old) (Siegel et al., 2019; Siegel et al., 2020b). Therefore, not only is it imperative that all aspects of its aetiology are considered, but also all potential options for treatment. Interestingly, viruses have been suggested as one such possible avenue, with the aim of exploiting the onco- and bacteriolytic properties of some viruses (Choi et al., 2016; Paule et al., 2018). In this article, we will explore the potential involvement of viruses in CRC aetiology, and examine how they could be applied in its mitigation.

## Viruses in Colorectal Cancer Aetiology

An array of viruses have been demonstrated to contribute to cancer development across the body. For example, human papillomavirus (HPV) infections are associated with cervical and oral cancers (Crosbie et al., 2013; Conway et al., 2018), hepatitis B and C viruses (HBV and HCV respectively) are linked with liver cancers (Lin et al., 2015; Xie, 2017), human herpes viruses are connected to the development of Kaposi sarcomas (Mariggiò et al., 2017), and Eppstein-Barr virus (EBV) is linked to nasopharyngeal cancers, Hodgkin’s lymphomas, and Burkitt’s lymphomas (Farrell, 2019). While the associations between these viruses and their respective cancers are well established, in CRC the involvement of viral infection in disease is less defined. Evidence does exist though — probably the most deeply examined connections involve members of the *Polyomaviridae* family, particularly the JC polyomavirus (JCPyV), although other viruses have also been linked to CRC, including HBV, HPV, and EBV (Su et al., 2020; Fernandes et al., 2020).

Polyomaviruses are regularly faced by humans, highlighted by the frequency with which antibodies targeting them are found within the population (Kean et al., 2009). Infection is normally asymptomatic, likely occurring following the consumption of virus-contaminated water or food (Bofill-Mas et al., 2001). Suggestions of their involvement in CRC began after JCPyV DNA was observed in colonic tumor samples. Although also found in the surrounding normal epithelia, JCPyV-loads were around 10-fold higher in cancerous tissues (Laghi et al., 1999). Subsequent studies corroborated these observations. Theodoropoulos et al. (2005) for example, found that 49/80 colon adenocarcinomas samples they examined were positive for JCPyV DNA, with mean viral load around 60-fold higher in carcinomas than in the adjacent non-cancerous epithelia. Other members of the *Polyomaviridae*, including BK polyomavirus and simian virus 40, have also been identified in CRC tumor tissue (Casini et al., 2005; Giuliani et al., 2008; Jarzyński et al., 2017).

**Figure 1:**
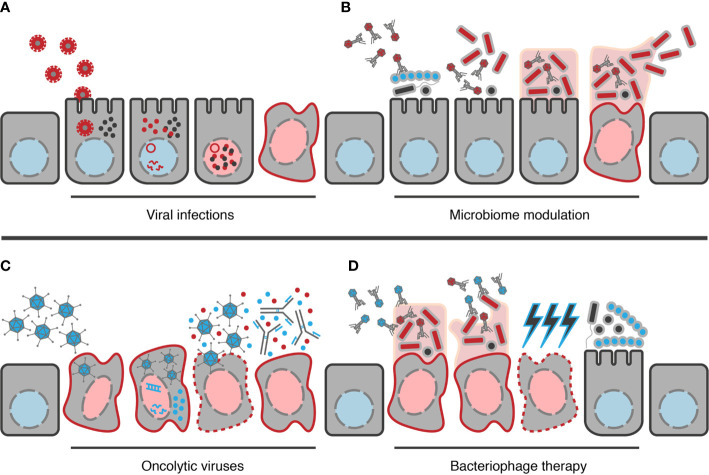
Examples of potential ways viruses can contribute to CRC development and how they could be applied in its treatment. **(A)** Example mechanism by which viral infections could contribute to disease, in this case polyomaviruses (red viruses). Following infection of the cell, viral DNA reaches the nucleus, leading to generation of viral transcripts and the production of viral proteins, e.g. T-Ag (red dots). These can interact with host effectors, e.g. β-catenin (black dots), resulting in changes in host gene expression from the healthy state (blue nucleus) to an oncogenic state (red nucleus). **(B)** Model example of how bacteriophages may contribute to cancer. Bacteriophages alter the composition of the gut bacterial community towards a configuration that allows colonization of oncogenic organisms (red bacteria). Following establishment, the oncogenic organisms can then form biofilms and begin to contribute towards transformation of the cell to a cancerous state. Once a cancerous state has developed, bacteria may enter a synergistic relationship with the cells, receiving peptides for their growth while continuing to contribute to maintenance of the cancerous state. In mature biofilms, bacteriophages will also contribute to dispersion of cells to establish similar cycles elsewhere. **(C)** Example of how oncolytic viruses (blue viruses) may be used in CRC treatment. Here, modified viruses are introduced proximal to the cancerous location, allowing them to locate and infect the target cells. When viral nucleic acid then reaches the nucleus, viral transcripts begin to be generated leading to production of progeny virions. If DNA sequences encoding therapeutic products have been inserted into the genome, e.g. GM-CSF (blue dots) here, these will be produced alongside viral proteins. Once assembled, progeny virions can then be released from the cell ***via*** lysis of the cancer cell, also causing the release of the therapeutic protein and antigens from the cancer cell itself (red dots); the latter of which can then stimulate the hosts immune response to target the cancer cell, possibly enhanced by the therapeutic product if it is an immunostimulatory product such as GM-CSF. **(D)** Model example of the use of bacteriophages therapeutically (blue bacteriophages) in CRC treatment. Bacteriophages are able to degrade biofilms through their depolymerases, providing access to the oncogenic organisms residing within. The bacteriophage can then infect and lyse these bacteria, selectively removing only the oncogenic organisms, reducing the oncogenic pressure on cells. As oncogenic bacteria can also confer chemoresistance with colonization, their removal may increase the sensitivity of the cancerous cell to chemotherapeutic treatments (blue lightning bolts). Selective removal of oncogenic organisms also clears the niche, allowing recolonization by other members of the microbiome or beneficial organisms introduced therapeutically.

The oncogenic potential of polyomaviruses is believed to stem mainly from their large T-antigen (T-Ag; **Figure 1A**). In the polyomavirus life cycle, T-Ag serves multiple purposes, including interacting with host proteins to alter cell cycle conditions and to replicate the viral genome (Eash et al., 2006). T-Ag can also interact with other cellular proteins though, including those involved in pathways related to CRC development. One example is β-catenin, a transcriptional activator and componentof the Wnt signalling pathway that, when in the nucleus, activates transcription factors associated with the proliferation of colonic epithelial cells and apoptosis (Zhang and Shay, 2017; Agrawal et al., 2019). When mutations occur that increase levels of β-catenin in the cell and the nucleus [e.g inactivation of the negative regulator adenomatous polyposis coli (*APC*)], increased transcription of key tumor progression genes results, such as c-*myc* and cyclin D1, leading to abhorrent cell growth and ultimately tumorigenesis (Powell et al., 1992; Morin et al., 1997; He et al., 1998; Tetsu and McCormick, 1999; Henderson, 2000). T-Ag can interact directly with β-catenin and facilitate its translocation into the nuclei, significantly increasing transcription of carcinogenic β-catenin targets (Enam et al., 2002; Ripple et al., 2014). *In vitro*, Ricciardiello et al. (2003) noted that exposure of colonic epithelial cell lines to JCPyV produced cells with aberrant chromosomes with signs of chromosomal breakages and aneuploidy, correlating with nuclear accumulation of β-catenin and T-Ag—chromosomal changes that are often observed in CRC (Bakhoum and Cantley, 2018). T-Ag has also been linked to metastasis, with one study finding T-Ag expression in >70% of metastatic primary tumors and their matching liver metastasis, and that T-Ag expression *in vitro* could increase cell migration (Link et al., 2009).

However, while some studies have found T-Ag expression in as many as 35–60% of CRC carcinomas examined (Enam et al., 2002; Nosho et al., 2009) and others have observed a significant association between its presence in CRC tissues and genetic changes commonly observed in CRC (Goel et al., 2006) the involvement of polyomaviruses in CRC remains controversial. For example, studies have been unable to detect its expression in any CRC samples, even when 26.1% of samples tested positive for T-Ag DNA (Hori et al., 2005). While, in other studies, little to no polyomavirus DNA was detected in CRC samples at all (Newcomb et al., 2004; Campello et al., 2010; Gock et al., 2020). Yet, there is the possibility that these observations could be justified by a temporal change in viral expression in what has been referred to as a “hit and run” style dynamic. In this dynamic, it is proposed an initial infection leads to the initiation of events that result in cellular transformation towards a cancerous phenotype (e.g. chromosomal breakages), but, after this event, viruses then face selection pressures that either alter gene expression levels or begin to select against viral presence altogether (Niller et al., 2011). Therefore in the case of polyomaviruses, viral infection and expression of T-Ag may trigger initial dysplasia but is no longer required or actively selected against in later disease. This idea is supported by observations showing equivalent JCPyV DNA loads in carcinomas as in adenomas, benign tumors of the epithelium that normally serve as the precursor for carcinomas, and where T-Ag expression can also be found (Theodoropoulos et al., 2005; Jung et al., 2008).

Regardless of whether viral infection is associated with CRC, viruses may still contribute to CRC in other ways, such as through their ability to alter microbial population structures. The gut microbiome is believed to play an important role in CRC aetiology. For example, studies have observed that the CRC intestinal microbiome is often characterized by reductions in butyrate-producing organisms, such as *Faecalibacterium* spp. and *Roseburia* spp., but is enriched for the likes of enterotoxigenic *Bacteroides fragilis* and *Escherichia coli*, or other pathobionts including *Fusobacterium* spp., *Selenomonas* spp., and *Porphyromonas* spp. (Chen et al., 2012; Wang et al., 2012; Wu et al., 2013; Hibberd et al., 2017). Members of the enriched sets of organisms are believed to promote cancer development in CRC. For example, enterotoxigenic *B. fragilis* and strains of *E. coli* harboring the polyketide synthase (*pks*) genomic island have been shown to produce tumorigenesis in murine models of CRC (Wu et al., 2009; Arthur et al., 2012). Evidence is particularly strong for the involvement of *Fusobacterium* spp. in CRC, in particular *Fusobacterium nucleatum*. *F. nucleatum* is commonly found as part of the oral microbiota of humans, as well as in other mucosal locations, where it serves important roles in community biofilm organization (Brennan and Garrett, 2019). In CRC, studies have found *Fusobacterium* spp. to be enriched within tumor sites relative to the surrounding non-tumorous tissue and that its abundance can correlate with patient survival, suggesting a link between this organism and the disease state (Kostic et al., 2012; Castellarin et al., 2012; Mima et al., 2016). Furthermore, when studied in isolation, colonization of *Apc* ^Min/+^ mice with *F. nucleatum* leads to the development of significantly more colonic tumors than that seen in untreated mice (Kostic et al., 2013). However, recently it has been suggested that the ability of such organisms to promote cancer may involve viral assistance.

In a study of the bacterial and viral populations in CRC, Hannigan et al. (2018) noted that there were strong associations between both the presence of specific bacterial “operational taxonomic units” (OTUs) and viral “operational viral units” (OVUs) with cancerous state; the OTU with the highest overall association to the cancerous state belonging to *Fusobacterium* spp. and the OVUs most strongly associated with disease belonging to bacteriophages—particularly the order *Caudovirales*. Nakatsu et al. (2018) also noted enrichment of bacteriophages in CRC faecal samples, and that, of 22 viral genera they found could maximally discriminate CRC samples from healthy controls, the majority were bacteriophages from the order *Caudovirales*. Hannigan and colleagues further observed that their OVU and OTU abundances were unrelated, leading them to examine if bacteriophages were “community hubs”, instead interacting with multiple organisms within the CRC microbiome. This was the case, with those bacteriophages interacting with the most organisms correlating positively with disease, suggesting that the bacteriophages acting as community hubs were important drivers of CRC. The authors then proposed a model of bacteriophage involvement in CRC wherein, bacteriophages with broad host-ranges can infect a wide range of bacteria within the gut microbiome, leading to initial dysbiosis (**Figure 1B**). This allows niche expansion of other untargeted organisms e.g. *F. nucleatum*, eventually resulting in the formation of a multi-species biofilm. Bacteriophages then assist within the biofilm through mechanisms such as contributing to nutrient/substrate availability and biofilm dispersal (Rossmann et al., 2015; Abedon, 2020). This provides a sufficient environment for bacteria to transform colonic epithelia, disrupt tight junctions, and infiltrate cells to produce a pro-cancer inflammatory response. Although not included in their model, Hannigan et al. (2018) do not exclude the possibility that direct interactions between bacteriophages and human cells may also contribute to the pro-cancer inflammatory responses seen in CRC. For example, members of the *Caudovirales* have been observed to directly interact with human cells (Lehti et al., 2017), cross epithelial barriers (Nguyen et al., 2017), and produce pro-inflammatory responses (Van Belleghem et al., 2017). Other bacteriophage taxa associated with CRC have also been shown to interact with the human host. Nakatsu and others observed Inovirus to be the genus of bacteriophage able to provide the greatest level of differentiation between CRC samples and the healthy controls in their study (Nakatsu et al., 2018). Members of this genus are known to play prominent roles in biofilm formation in some bacteria (Secor et al., 2015), decorate themselves with potentially immunogenic bacterial proteins (Piekarowicz et al., 2020), and even directly alter immune responses (Sweere et al., 2019).

There remain many other unknowns relating to the virome and disease in CRC that require further study to decipher. To give one important example, Nakatsu and colleagues found that the viral genus with the greatest discriminatory capacity between healthy and CRC samples was not a bacteriophage, but rather *Orthobunyavirus*, a viral taxon with no previously described role in human gastrointestinal disease (Nakatsu et al., 2018). Thus future studies must be conducted to resolve the relations between gut viral populations and CRC aetiology. These studies face considerable challenges though. Virome studies, for instance, must contend with considerable inter-individual variability (Shkoporov et al., 2019). Given that virome variability can be influenced by factors such as geography, diet, and ethnicity (Minot et al., 2011; Zuo et al., 2020), these studies should be longitudinal and recruit large numbers of individuals from diverse origins, cultures, and ethnic backgrounds. Recruitment of these large cohorts then will be in and of itself a considerable hurdle to overcome. Viromes also contain a vast amount of so-called “dark matter”—sequences with no known origin. This can account for as many as 90% of the reads within some virome data sets (Aggarwala et al., 2017). As such, viral isolation studies are also needed to assign a viral origin to the unknown sequences. This may require the development of new viral isolation methodologies for those viruses unable to be isolated through standard procedures. Methodological changes in bacterial isolation protocols have allowed “unculturable” bacteria previously only seen in metagenomic samples to be isolated and characterized (Browne et al., 2016). Overcoming these hurdles is no small feat, however, the potential clinical benefits are significant. Given that the existing studies of the CRC virome suggest the virome can discriminate between healthy individuals and those with CRC, and even predict both disease state and prognosis (Hannigan et al., 2018; Nakatsu et al., 2018), and that these associations are likely a reflection of underlying pathophysiological events, further insights could lead to important advances in both CRC diagnostics and treatment.

## Viruses in Colorectal Cancer Treatment

Viruses have a long history relating to the treatment of cancer, stemming from a series of reports where patients displayed periods of spontaneous remission after viral exposure. In an 1897 report, a patient previously diagnosed with myelogenous leukaemia was observed to undergo temporary disease regression following a believed influenza infection (Dock, 1904). Numerous accounts in the years that followed also reported improvements in patients after either viral infections or vaccination (e.g. De Pace (1912); Bierman et al. (1953); Bluming and Ziegler (1971); Hansen and Libnoch (1978)). This led viruses to be examined as potential therapeutic options against cancers. In an early example, after observing two cases where patients with Hodgkin’s lymphoma underwent a period of remission after viral hepatitis, Hoster et al. (1949) examined the effects of introducing sera and tissue containing the hepatitis virus to 21 Hodgkin’s lymphoma patients. Their tests bore success, albeit temporary, observing signs of improvement for at least one month in 7 of 13 patients in whom viral hepatitis developed. Today, viruses are still being examined for their therapeutic uses in cancers, including those akin to these early studies, namely oncolytic viruses (OVs).

The therapeutic benefits of OVs against cancers are two-fold. Firstly, OVs are able to preferentially infect cancerous cells, culminating in their lysis. Secondly, lysis of the infected cell results in the release of tumor antigens and viral particles into the surrounding environment, stimulating the host’s immune system and generating a complementary host-derived anti-cancer response (Melcher et al., 2011; Chiocca and Rabkin, 2014). Although early anti-cancer studies utilized wild-type viruses, oncolytic viral therapies have since been refined utilising our expanded knowledge of the underlying disease and advancements in molecular biology. This has allowed us to modify viruses to improve their safety and efficacy. Modifications can be in the form of mutations that broaden viral tropisms, remove unwanted characteristics, enhance immunogenicity, or any of a series of other therapeutically beneficial genetic alteration (Choi et al., 2016; Zheng M. et al., 2019).

One well-known example of a therapeutically modified virus is the talimogene laherparepvec (T-VEC) system (**Figure 1C**). T-VEC is a herpes simplex type-1 virus (HSV-1) that has been approved by both the US Food and Drug Administration (FDA) and the European Medicines Agency (EMA) for use in the treatment of metastatic melanomas but is also currently being trialed for use in CRC (Mullard, 2015; Raman et al., 2019). Three noteworthy modifications have been introduced in this system (Liu et al., 2003). The first involves the deletion of *ICP34.5*, which is associated with neurovirulence and antiviral evasion, to improve safety (Chou et al., 1990; Tallóczy et al., 2002; Orvedahl et al., 2007). While the safety benefits of reducing neurovirulence are self-explanatory, the removal of antiviral evasion improves safety by increasing selectivity of T-VEC towards cancer cells. *ICP34.5* allows viral antagonism of protein kinase R (PKR), an important antiviral defence in healthy cells that is often dysfunctional in cancer cells (Kohlhapp and Kaufman, 2016; Gal-Ben-Ari et al., 2019). Removal of *ICP34.5*, therefore, attenuates antagonism in healthy cells, while in cancer cells viral infection can proceed despite the deletion. The second modification of T-VEC is the deletion of *ICP47*, a gene which allows HSV to reduce the hosts immune detection of infected cells by inhibiting antigen presentation (Hill et al., 1995). Its removal allows antigen presentation to occur, improving efficacy. Deletion of *ICP47* also leads to the increased expression of *US11* by shifting its expression to the control of the *ICP47* promoter, which has been shown to improve viral growth in cancer cells with no impact to safety (Taneja et al., 2001; Liu et al., 2003). Lastly, two copies of the human granulocyte-macrophage colony-stimulating factor gene, *GM-CSF*, have been added to the T-VEC system, which recruits and activate antigen-presenting cells to the local area and induces anti-tumor T-cell responses (Liu et al., 2003; Kaufman et al., 2014).

Other viruses are also being examined in clinical trial for use in CRC, with various different modifications. Examples include ONYX-015, an adenovirus that has been altered to remove the viral *E1b* gene (reducing its ability to infect non-cancerous cells) (Heise et al., 1997; Reid et al., 2001); Pexa-Vec (pexastimogene devacirepvec; JX-594), a vaccinia virus also modified to encode GM-CSF, but with the additional modifications to inactivate the viral thymidine kinase gene (reducing its ability to infect non-cancerous cells) and to add encoding of β-galactosidase (as a reporter) (Kim et al., 2006; Park et al., 2015); and even an adenovirus, Enadenotucirev (EnAd; ColoAd1), where modifications have not been directly introduced but rather are the result of recombination between different adenovirus serotypes following the passage of mixed viral pools through cancer cell lines (in this case HT-29 cells) in a process termed “Directed Evolution” (Kuhn et al., 2008; O’Cathail et al., 2020). Unmodified viruses, such as the reovirus Pelareorep (Reolysin), are also being examined in clinical trials for use in CRC treatment (Goel et al., 2020).

While the application of OVs could be useful in the treatment of cancer itself, there remains the issue of pro-cancer antagonism from members of the surrounding bacterial population, such as that seen for *F. nucleatum* (Kostic et al., 2013; Wu et al., 2019). Therefore, removal of the pro-CRC organisms would also be of therapeutic benefit. Bullman et al. (2017) examined the impact of antibiotic treatment on CRC tumor growth in a murine model using human CRC xenografts that were heavily associated with *Fusobacterium* spp. and found that antibiotic treatment reduced the rate of tumor cell proliferation and overall tumor growth. However, while antibiotic therapies could be a simple means of removing oncogenic organisms, the breadth of their action is not restricted to pro-cancer organisms (Lange et al., 2016). Other members of the colonic bacterial population can actually be anti-carcinogenic, and their collateral removal could have deleterious ramifications. For example, some bacteria can produce metabolites with anti-cancer properties following the fermentation of dietary fibres. One example is butyrate, a product of bacterial fermentation that is believed to protect the gut against development of CRC (Fung et al., 2012). In one study, colonization of BALB/c mice with *Butyrivibrio fibrisolvens*, a bacteria that can produce butyrate following fermentation of fibre, was shown to protect mice with high fibre diets more from CRC development than those mice without the bacteria (Donohoe et al., 2014). For this reason, a more selective approach to antimicrobial treatment in CRC would be desirable and could be achieved through the therapeutic use of bacteriophages (**Figure 1D**).

Bacteriophage-based therapeutics already exist and have existed for around 100 years (Sulakvelidze et al., 2001). Bacteriophage therapy (also known as phage therapy) is the most widely studied therapeutic application involving bacteriophages and describes the application of whole bacteriophage particles to treat bacterial infections (Kakasis and Panitsa, 2019). Although not common in Western medicine, bacteriophage therapy has been in use in Eastern Europe for decades, particularly in Georgia, where the Eliava Institute of Bacteriophages, Microbiology, and Virology has been examining their therapeutic potential since 1923 (Kutateladze, 2015). In the last two decades, the reinvigorated interest in bacteriophage therapy has primarily been motivated by the emergence of antibiotic-resistant microorganisms, for which it has been deployed successfully against in a number of high-profile examples [e.g. Schooley et al. (2017); Dedrick et al. (2019)]. Recently though, interest has grown for its application in microbiome manipulation, largely due to the specificity of bacteriophages (Lee et al., 2018). Bacteriophages can have small host-ranges, often only infecting a minute subset of strains within a particular species, negating the issue of indiscriminate action seen with broad-spectrum antibiotics (Flores et al., 2011; Ganeshan and Hosseinidoust, 2019). Bacteriophages also often encode depolymerases that allow them to degrade biofilms and gain access to the residing organisms (Alves et al., 2016; Knecht et al., 2020). *Fusobacterium* spp. are often found in biofilms in CRC, with this form of community structure believed to be important in the promotion of tumorigenesis (Dejea et al., 2014; Flynn et al., 2016; Bullman et al., 2017). Much like their oncolytic relatives, bacteriophages can also be engineered to carry additional therapeutic benefits (Kilcher and Loessner, 2019), which has been examined in CRC.

Zheng D. W. et al. (2019) examined the impact of a “bacteriophage-guided biotic–abiotic hybrid nanosystem” as a treatment option in mice bearing CT26 colorectal carcinomas. In this system, bacteriophages targeting *Fusobacterium* spp. are utilized along with two abiotic components; irinotecan, a first-line treatment for colorectal cancer, and dextran, a fermentable complex glucan (Olano-Martin et al., 2000; Fuchs et al., 2006). The bacteriophages are azide-modified, while irinotecan is encapsulated within azodibenzocyclooctyne-modified dextran nanoparticles. These modifications mean the irinotecan-dextran nanoparticles can link covalently to the bacteriophages, allowing the nanoparticles to concentrate within areas where *Fusobacterium* spp. are present, i.e. within the tumor sites. Irinotecan is used for its chemotherapeutic properties, while dextran is employed as a prebiotic due to the ability of fermentable carbohydrates to promote shifts in microbial colonization and metabolite production (Harris et al., 2017; Warren et al., 2018). Here, the use of dextran aimed to increase the abundance of butyrate and butyrate-producing bacteria within the gastrointestinal tract because of their anti-tumorigenic properties (Fung et al., 2012). It was observed that their bacteriophage-guided nanosystem was able to significantly decreases *Fusobacterium* spp. levels, increase butyrate production, and importantly, suppress tumor growth.

Although viral therapies do show considerable promise they do have their drawbacks (Loc-Carrillo and Abedon, 2011; Zheng M. et al., 2019). In the case of bacteriophage therapy, although some studies have shown that the introduction of bacteriophages can have only minimal or transient effects on non-target bacterial populations (Reyes et al., 2013; Nale et al., 2018), other studies have shown that the impact could be broader (Hsu et al., 2019; Lange et al., 2019). There is also the issue of bacterial resistance development, and although currently this is mainly overcome by the use of bacteriophage “cocktails” (mixes containing multiple distinct bacteriophages; Nale et al. (2016)), resistance can still occur, meaning co-use with antibiotics can be required to completely eliminate the target organism (Oechslin et al., 2016). The ability to modify viruses though could allow us to overcome many of these issues (Twumasi-Boateng et al., 2018; Kilcher and Loessner, 2019). For example, in this latter case, alternative antimicrobials [e.g. silver nanoparticles; Dong et al. (2020)] could be attached to the bacteriophage particles to provide an additional means of locally-contained antibacterial action against resistant cells. As with the involvement of viral disease in CRC aetiology though, it should be echoed that much remains to be learned about host and microbiome dynamics within CRC, in particular at the individual level (Panagi et al., 2019). Further investigations are warranted to ensure laboratory promise can be translated to clinical success as although trials of viral therapies to treat other conditions and other cancers have brought success (Wright et al., 2009; Andtbacka et al., 2015), success has not been ubiquitous (Sarker et al., 2016; Guo, 2020).

Despite these points, viral therapeutics are an avenue worth exploring with huge potential through molecular engineering. One exciting proposition would be an “anti-cancer viral cocktail” combining engineered OVs and bacteriophages. This would allow the benefits of both oncolytic targeting of cancerous cells and promotion of anti-cancer immune responses from the OVs, while also providing the benefit of the antimicrobial action of bacteriophages. The viruses themselves could be modified to provide further benefits, be it increased immunogenicity through the addition of immunostimulatory products such as GM-CSF to the OVs, or addition of pre-biotics and chemotherapeutics to bacteriophages allowing targeted delivery to the site of the tumor, promoting the growth of beneficial organisms and impeding tumor growth. Whatever shape the desired modifications take, when it comes to viral therapies, the possibilities are endless.

## Author Contributions

All authors were involved in the conception of the review topic and focus. CT and AV contributed to the initial draft of the manuscript. CT generated the illustrations. All authors contributed to the article and approved the submitted version. 

## Funding

This work was supported by startup research funds provided by the University of California Merced School of Natural Sciences.

## Conflict of Interest

The authors declare that the research was conducted in the absence of any commercial or financial relationships that could be construed as a potential conflict of interest.
